# Azithromycin decreases NALP3 mRNA stability in monocytes to limit inflammasome-dependent inflammation

**DOI:** 10.1186/s12931-017-0608-8

**Published:** 2017-06-28

**Authors:** Elizabeth A. Lendermon, Tiffany A. Coon, Joseph S. Bednash, Nathaniel M. Weathington, John F. McDyer, Rama K. Mallampalli

**Affiliations:** 1 Pulmonary, Allergy, & Critical Care Medicine, Department of Medicine, University of Pittsburgh, UPMC Montefiore, NW 628, Pittsburgh, PA 15213 USA; 2 Acute Lung Injury Center of Excellence, University of Pittsburgh, Pittsburgh, PA USA; 3 Medical Specialty Service Line, Veterans Affairs Pittsburgh Healthcare System, Pittsburgh, PA USA

## Abstract

**Background:**

Azithromycin, an antibiotic used for multiple infectious disorders, exhibits anti-inflammatory effects, but the molecular basis for this activity is not well characterized. Azithromycin inhibits IL-1β-mediated inflammation that is dependent, in part, on inflammasome activity. Here, we investigated the effects of azithromycin on the NACHT, LRR, and PYD domains-containing protein 3 (NALP3) protein, which is the sensing component of the NALP3 inflammasome, in human monocytes.

**Methods:**

THP-1 cells were treated with azithromycin alone, LPS alone, or both. NALP3 and IL-1β protein levels were determined by immunoblotting. NLRP3 gene (encoding NALP3) transcript levels were determined by quantitative qPCR. In order to measure NLRP3 transcript decay, actinomycin D was used to impair gene transcription. THP-1 Lucia cells which contain an NF-κB responsive luciferase element were used to assess NF-κB activity in response to azithromycin, LPS, and azithromycin/LPS by measuring luminescence. To confirm azithromycin’s effects on NLRP3 mRNA and promoter activity conclusively, HEK cells were lipofected with luciferase reporter constructs harboring either the 5’ untranslated region (UTR) of the NLRP3 gene which included the promoter, the 3’ UTR of the gene, or an empty plasmid prior to treatment with azithromycin and/or LPS, and luminescence was measured.

**Results:**

Azithromycin decreased IL-1β levels and reduced NALP3 protein levels in LPS-stimulated THP-1 monocytes through a mechanism involving decreased mRNA stability of the NALP3 – coding NLRP3 gene transcript as well as by decreasing NF-κB activity. Azithromycin accelerated NLRP3 transcript decay confirmed by mRNA stability and 3’UTR luciferase reporter assays, and yet the antibiotic had no effect on NLRP3 promoter activity in cells containing a 5’ UTR reporter.

**Conclusions:**

These studies provide a unique mechanism whereby azithromycin exerts immunomodulatory actions in monocytes by destabilizing mRNA levels for a key inflammasome component, NALP3, leading to decreased IL-1β-mediated inflammation.

## Background

Macrolides are a group of antibiotics that are used widely in inflammatory pulmonary diseases for known immunomodulatory effects that appear to be distinct from their antimicrobial effects. Azithromycin, a macrolide, has been shown to decrease exacerbations in COPD [1], improve lung function in cystic fibrosis [2–5], improve lung function in diffuse panbronchiolitis [6–8], and decrease the rate of decline of lung function in bronchiolitis obliterans syndrome after lung transplantation [9–12]. In addition, there is mounting interest in employing the anti-inflammatory activity of macrolide antibiotics to complement other treatment modalities with bacteriocidal, bacteriostatic, or even anti-viral actions. Recently, azithromycin’s effect on inflammation was demonstrated in hospitalized patients with influenza as an adjuvant to oseltamivir [13]. Many of these disorders are mediated, in part, by actions of ﻿interleukin 1β (IL-1β) elaborated by the inflammasome. Thus, understanding the molecular mechanisms of azithromycin’s activity in the inflammasome may be critical in devising newer macrolide analogs with even greater potency to better target deleterious pathways. However, these mechanisms by which azithromycin exerts beneficial effects in inflammatory diseases have not been characterized fully.

Azithromycin is known to inhibit activity of a master transcriptional mediator of inflammation, nuclear factor kappa B (NF-κB), in multiple experimental models. The compound inhibits the nuclear binding activity of NF-κB in macrophages in response to both endotoxin (lipopolysaccharide [LPS]) and reactive oxygen species in vitro [14, 15]. Additionally, azithromycin reduces NF-κB DNA binding in a cystic fibrosis airway epithelial cell line [16], and it suppresses *Acintobacter baumannii*-induced nuclear translocation of NF-κB in human airway epithelial cells [17]. Azithromycin has also been shown to decrease NF-κB activation in vivo in a mouse model of LPS-induced pulmonary inflammation [18]. Nonetheless, the mechanism by which azithromycin inhibits NF-κB activation is incompletely characterized, and alternative regulatory activity of azithromycin in inflammation has not been extensively explored.

Azithromycin also appears to down-regulate expression of inflammatory cytokines downstream of NF-κB. Azithromycin attenuates LPS-induced pulmonary neutrophilia by inhibiting IL-1β expression in alveolar macrophages [19, 20], and the compound also decreases levels of IL-1β in a murine model of lung ischemia reperfusion injury [21]. While, NF-κB activation is necessary for the gene transcription of inactive forms of inflammatory cytokines such as pro-IL-1β (as well as pro-IL-18), cleavage by caspase-1 is required for generation of their active forms. Inflammasomes are multimeric protein complexes that directly recruit and activate caspase-1 and therefore generate active IL-1β. Only recently have there been investigations of the effects of azithromycin on inflammasome activity. Gualdoni et al. showed that azithromycin decreases induction of caspase-4, an early regulator of the inflammasome cascade, in human monocytes [22].

Since inflammasome activity is critical in the generation of active IL-1β which is downregulated by azithromycin in multiple experimental models of inflammation, we hypothesize that inflammasome inhibition may be a distinct and complementary mechanism of azithromycin’s negative effect on inflammation. Here, we investigated the effects of azithromycin on the NLRP3 gene transcript, as well as its translated protein, the NACHT, LRR, and PYD domains-containing protein 3 (NALP3) which functions as the sensing component of the NALP3 inflammasome. We present data that suggest that azithromycin reduces NALP3 driven IL-1β release in cells by a unique ability to destabilize NLRP3 mRNA levels and thus decrease cells transcriptional response to endotoxin.

## Methods

### Cells

THP-1 cells (Cat #TIB-202 from ATCC, <passage 20) were cultured in Roswell Park Memorial Institute (RPMI) 1640 medium supplemented with 10% fetal bovine serum (FBS) and antibiotics. HEK cells (Cat #CRL-1573 from ATCC, <passage 20) were cultured with Dulbecco's modified Eagle's medium (DMEM) containing 10% FBS and antibiotics. Peripheral blood mononuclear cells from two healthy donors were purchased as leukopaks from a blood bank.

### Chemicals used

The following reagents were used: LPS (Sigma), azithromycin (Fresensius Kabi USA, LLC) at concentrations indicated, and actinomycin D (Sigma) at 5 μg/ml.

### Immunoblotting

Briefly, cells were lysed in RIPA buffer supplemented with protease inhibitors (Thermo Scientific), and lysates were sonicated and then centrifuged to remove debris. Equal amounts of total protein in sample buffer were loaded on SDS-polyacrylamide gels and processed for immunoblotting using the following antibodies: NALP3 (Adipogen AG-20B-0014, 1:1000), NALP6 (Abcam ab58705, 1:1000), Nalp7 (Abcam ab126979, 1:2000), Pro-IL-1β (R&D Systems clone 615417, 1:1000), caspase-1 (R&D Systems mAB615, 1:1000), and β-actin (Sigma clone AC-15, 1:10,000). Immunoblots were exposed to SuperSignal West Femto chemiluminescent substrate (Thermo Scientific).

### qRT-PCR

RNA was isolated from THP1 cells using the RNeasy Mini Kit from Qiagen (Valencia, CA) per the protocol supplied in the kit. The concentration of each RNA sample was measured, followed by conversion to cDNA using the High-Capacity RNA-to-cDNA kit from Thermo Fisher Scientific using 20 ng of mRNA to generate cDNA for each sample. RNA quality was evaluated with nanoDrop, and all samples used for qPCR had both 260/230 and 260/280 ratios above 1.8. Real-time PCR was carried out in a C1000 Thermal Cycler from Bio-Rad (Hercules, CA) using SYBR Select Master Mix from Thermo Fisher Scientific per the included protocol. The primers used were NLRP3 (5′-ATGAGTGCTGCTTCGACATC-3′, 5′-TTGTCACTCAGGTCCAGCTC-3′), and GAPDH (5′-ATCATCCCTGCCTCTACTGC-3′, 5′-GTCAGGTCCACCACTGACAC-3′). Primers were designed using the NLM NCBI Primer design tool with stringency to elaborate a 100–300 bp amplicon that spanned an exon junction of the target gene and included at least 2 mismatches when interrogated against all genes for unintended targets. We used GAPDH as a reference gene by standard methods. This is an appropriate housekeeping gene based on our prior experience [23]. There was no observed effect of azithromycin treatment on GAPDH gene levels. The ddCq (ΔΔCq) method was used to determine fold change. In some experiments, fold change was used to calculate percent mRNA remaining using the following equation: % remaining = Fold change × 100.

### Plasmids and transfection

LightSwitch Promoter and 3’UTR Reporter GoClone plasmid DNA constructs for the NLRP3 gene were purchased from Switchgear Genomics (Product ID S718266 and S805599). HEK cells were transfected with these plasmids and an empty plasmid as per manufacturer protocol using FuGene HD Transfection Reagent (Promega). Lightswitch Luciferase Assay Reagent (Switchgear Genomics) was used prior to luminometry to measure luciferase reporter signals also per manufacturer protocol.

### THP-1-Lucia NF-κB Cells

Cells were purchased from InvivoGen. Luciferase reporter signals were measured using Quanti-Luc reagent (InvivoGen) according to manufacturer protocol prior to luminometry.

### Statistics

One and two way ANOVA were used for statistical analysis for comparison of multiple groups. Dunnett’s and Bonferroni’s multiple comparison tests were used for post-hoc analysis. Data are shown as means with standard error of the mean.

## Results

### Azithromycin decreases NALP3 expression and IL-1β in THP-1 monocytes

**Fig. 1:**
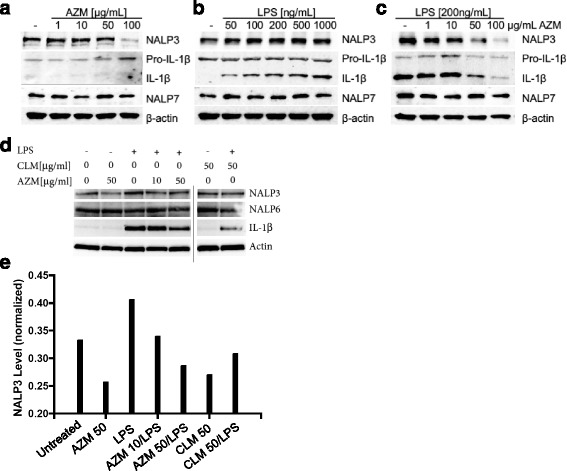
Azithromycin Decreases NALP3 Protein in THP-1 Monocytes. **a** THP-1 cells were treated with azithromycin at concentrations shown for 12 h overnight prior to immunoblot analysis for indicated proteins. **b** THP-1 cells were plated and rested overnight. Cells were treated with LPS at concentrations shown for 4 h prior to immunoblot analysis. **c** THP-1 cells were treated with azithromycin at concentrations shown for 12 h overnight and then treated with LPS for 4 h prior to immunoblot analysis. The data representative of 2–3 separate experiments. **d** Peripheral blood mononuclear cells were treated with azithromycin or clarithromycin at concentrations shown for 12 h overnight. Plate-adherent monocytes were then treated with LPS 500 ng/ml for 4 h prior to lysis and immunoblot analysis for indicated proteins. Data shown is representative of multiple experiments. **e** NALP3:actin densitometry of immunoblot shown in Figure D

**Fig. 2:**
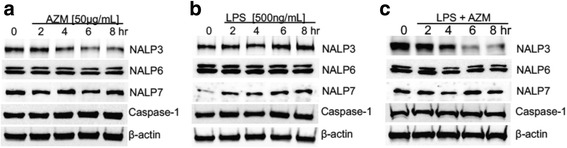
Kinetics of Azithromycin Effects on NALP3 Protein in THP-1 Monocytes. **a** THP-1 cells were treated with azithromycin 50ug/ml for the times shown prior to immunoblot analysis for indicated proteins. **b** THP-1 cells were treated with LPS 500 ng/ml for times shown prior to analysis. **c** THP-1 cells were treated with azithromycin (50ug/ml) and LPS (500 ng/ml) for times shown prior to immunoblot analysis. The data representative of 2–3 separate experiments

The prior observation that azithromycin inhibits IL-1β secretion from monocytes [22] led us to evaluate the effects of this antibiotic on a critical inflammasome component, NALP3, in THP-1 monocytes. We treated THP-1 cells with a range of azithromycin concentrations for 12 h and evaluated NALP3 protein levels. As shown in Fig. 1a, azithromycin at 100 μg/ml decreased NALP3 protein levels under native, unstimulated, conditions in THP-1 monocytes. To assess the effects of azithromycin on NALP3 induction after stimulation, we exposed THP-1 cells to LPS for 4 h to induce measurable inflammatory responses (Fig. 1b). LPS stimulation produced a modest increase in NALP3 and IL-1β protein levels at a concentration of 200 ng/ml and higher (Fig. 1b). This was accompanied by cleavage of IL-1β to its active isoform, implicating inflammasome activation as an LPS response in these cells. Interestingly, these results of LPS in THP-1 cells differ from results in U937 cells where we observed a more robust increase in NALP3 protein mass with only a modest increase in NALP3 mRNA [24]. Next, we pretreated THP-1 cells for 12 h with escalating concentrations of azithromycin followed by 4 h of LPS stimulation (200 ng/ml) and measured NALP3 and IL-β levels (Fig. 1c). Azithromycin at or above a concentration of 50 μg/ml substantially decreased NALP3 and IL-1β levels in LPS-stimulated THP-1 monocytes. In order to determine whether this azithromycin effect on NALP3 protein level was also present in primary human monocytes, peripheral blood mononuclear cells were plated and treated for 12 h with escalating doses of azithromycin. The plate bound cells (monocytes) were then stimulated with LPS for 4 h prior to lysis and immunoblot analysis. As shown in Fig. 1d and e, azithromycin also decreased NALP3 and IL-1β levels in these primary human monocytes. We also pretreated some peripheral blood mononuclear cells with clarithromycin (50 μg/ml) to further investigate whether this decrease in NALP3 protein was specific to azithromycin or a class effect of macrolides. Interestingly, clarithromycin also appeared to decrease NALP3 and IL-1β protein levels in primary human monocytes suggesting a possible class effect. In assessing the kinetics for these anti-inflammatory effects of azithromycin, the compound decreased NALP3 expression in unstimulated THP-1 cells treated with azithromycin by 4–6 h and LPS modestly increased NALP3 mass by 4–8 h (Figs. 2a and 2b). Of note, effects of azithromycin were relatively selective for NALP3, as limited if any effects of the antibiotic were observed on steady-state pro-IL-1β, caspase-1, NALP7, or NALP6 levels (Figs. 1 and 2). Importantly, azithromycin potently reduced NALP3 protein levels in LPS-stimulated THP-1 monocytes by 4–6 h (Fig. 2c). Hence, these data indicate that azithromycin substantially limits expression the active form of the pro-inflammatory cytokine (IL-1β), by reducing abundance of the inflammasome component NALP3.

### Azithromycin decreases NLRP3 mRNA by decreasing transcript stability

**Fig. 3:**
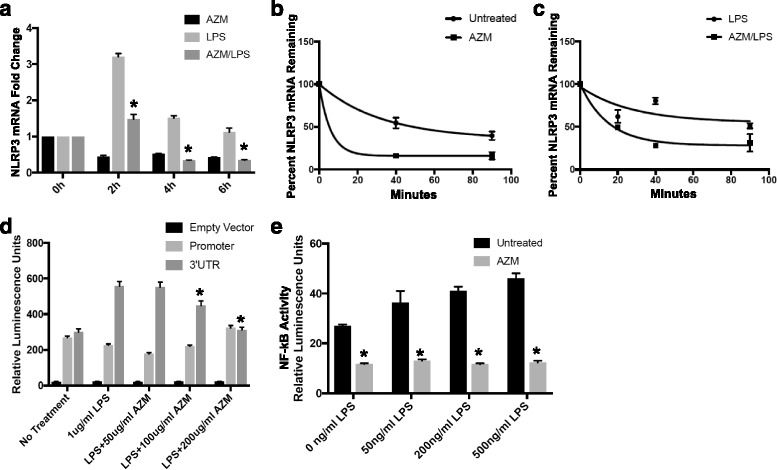
Azithromycin Decreases NLRP3 mRNA Stability and NF-kB Activity. **a** THP-1 cells were treated with azithromycin (50ug/ml), LPS (500 ng/ml), or both for the times shown prior to mRNA isolation and qPCR for relative NLRP3 mRNA measurement. *n* = 3. F (2,15) = 6.571, *p* = 0.009. **b** THP-1 cells were treated with azithromycin (50 ng/ml) for 6 h prior to adding actinomycin D (5ug/ml) to impair gene transcription. mRNA fold change was determined using qPCR. Percent mRNA remaining was calculated using the equation: (1 – ddCq) × 100. **c** THP-1 cells were treated with azithromycin (50 ng/ml) for 6 h and LPS (500 ng/ml) for 4 h prior to adding actinomycin D (5ug/ml). mRNA was quantified using qPCR. **d** HEK cells were lipofected with either plasmids containing an empty vector, plasmids containing a NLRP3 promoter region, or plasmids containing the 3’UTR of the NLRP3 overnight. All plasmids were linked to luciferase reporter constructs. Cells were then treated with azithromycin at concentrations shown for 1 h prior to addition of LPS (1ug/ml) for 4 h (untreated and LPS alone served as controls), and luminometry was used to measure luciferase activity. Data is representative of multiple experiments. A significant effect of azithromycin on luminescence in the cells transfected with 3’UTR was found, F (3,28) = 23.52, *p* < 0.0001. Dunett’s multiple comparisons test revealed a significant difference between 3’UTR luminescence in LPS-stimulated cells treated with no azithromycin and LPS-stimulated cells treated with azithromycin 100 μg/ml and 200 μg/ml (*p* = 0.0077 and *p* = 0.0001). **e** THP-1 Lucia cells (with NF-kB responsive luciferase construct) were treated with LPS alone at concentrations shown or LPS and azithromycin 50ug/ml for 12 h. NF-kB activity was quantitated by measuring luminescence. *n* = 3. A significant effect of azithromycin on luminescence was found, F (1,16) = 335.8, *p* < 0.0001. Bonferroni’s multiple comparison’s test also revealed significant *p* values for this azithromycin effect with all doses of LPS (*p* = 0.0002 with 0 ng/ml LPS, *p* < 0.0001 with 50 ng/ml LPS, *p* < 0.0001 with 200 ng/ml LPS, *p* < 0.0001 with 500 ng/ml LPS). There was also a significant effect of LPS on luminescence, F (3, 16), *p* = 0.0011

To determine if azithromycin reduces NALP3 protein at the pre-translational level, we assayed levels of its coding NLRP3 mRNA. We treated THP-1 monocytes with azithromycin 50 μg/ml, LPS 500 ng/ml, or both agents and isolated mRNA after 0–8 h of treatment and quantified transcripts using qPCR (Fig. 3a). In one sample of untreated THP cells, NLRP3 mRNA Cq signals reach a threshold by a mean of 7.46 cycles (dCq) behind that of GAPDH (mean Cq values of 28.96 vs 23.76). NLRP3 abundance is calculated as ddCq which is 0.027 fold that of GAPDH in unstimulated conditions and that quantity was normalized to 1 or 100% NLRP3 mRNA in Fig 3a. Azithromycin significantly decreased NLRP3 mRNA levels at all time points in LPS-stimulated cells (Fig. 3a). When cells were pretreated with azithromycin for 6 h, the dCq was increased by an average of 1.62 cycles (*n* = 3 separate experiments, data not shown). Next, to ascertain whether azithromycin exerts effects on NLRP3 mRNA stability, we performed experiments using actinomycin D to inhibit gene transcription and to allow the measurement of the rate of decay of the transcript over time. Unstimulated THP-1 cells treated with 50 μg/ml azithromycin for 6 h prior to the addition of actinomycin D showed a rapid decay of NLRP3 mRNA with a change in dCq of 1.13 in untreated cells after 60 min exposure (53.5% decrease), compared to dCq change of 1.96 in cells treated with azithromycin over the same time (74.3% decrease), corresponding to the relatively accelerated mRNA decay shown in Fig. 3b. Notably, THP-1 monocytes exposed to both LPS at 500 ng/ml and azithromycin at 50 μg/ml prior to addition of actinomycin D also displayed reduced NLRP3 mRNA abundance (Fig. 3c). Thus, these data demonstrate a mechanism by which azithromycin modulates NALP3 protein levels by destabilizing the NLRP3 transcript.

### Azithromycin increases NLRP3 mRNA degradation without altering NLRP3 promoter activity

Having shown an effect of azithromycin on NLRP3 mRNA stability, we next confirmed this finding using luciferase reporter constructs of the NLRP3 gene. These fragments contained a luciferase gene abutted by either the 5’ untranslated region (UTR) that includes the NLRP3 promoter (to assess the antibiotic’s effect on gene transcription) or the 3’ UTR of the gene (which contains regulatory sequences that may impact mRNA stability). In these experiments HEK cells were transfected overnight with a luciferase only - containing vector, plasmid luciferase constructs containing the NLRP3 promoter region, or luciferase constructs with the 3'UTR of NLRP3. Cells were then treated with azithromycin at concentrations shown for 1 h prior to the addition of LPS (1ug/ml) for 4 h (untreated and LPS alone served as controls), and luciferase activity was measured (Fig. 3d). The results demonstrated that azithromycin had no significant effect on NLRP3 promoter reporter activity. However, NLRP3 mRNA 3’UTR activity was significantly, but only partly decreased by azithromycin at concentrations of 100 μg/ml and 200 μg/ml. These data further support that azithromycin decreases NLRP3 mRNA stability; however, the inability of the antibiotic to totally abrogate NLRP3 mRNA 3’UTR activity in response to LPS may be related to the experimental conditions used or other regulatory response elements that are not contained within the fragment tested.

### Azithromycin decreases NF-κB activity in THP-1 cells

Prior studies in multiple experimental models suggest that NF-κB activity, a molecular input to IL-1β synthesis and release, is decreased by azithromycin [17]. Thus, in separate studies we assessed ability of azithromycin to inhibit NF-κB activation in THP-1 monocytes. Here, using THP-1 Lucia cells which contain an NF-κB responsive luciferase element, we exposed cells to LPS at escalating doses with and without 50 μg/ml azithromycin and assessed NF-κB activity by measuring luminescence. Azithromycin significantly decreased mRNA stability by ~50–62% demonstrating that in addition to reduction of steady-state NLRP3 mRNA, the antibiotic also suppresses a canonical pathway integral to cytokine gene expression (Fig. 3e). These data suggest that azithromycin decreases IL-1β expression in monocytes by multiple mechanisms.

## Discussion

Several studies have demonstrated beneficial anti-inflammatory effects of macrolides in pulmonary diseases that are independent of any antimicrobial activity. Here, we sought to further evaluate the effects of azithromycin specifically on the NALP3 inflammasome. We have shown that azithromycin within several hours inhibits both NALP3 and IL-1β expression in LPS-stimulated human monocytes in a dose-dependent fashion. Additionally, we show for the first time that NALP3 protein expression is decreased by an azithromycin effect at the mRNA level, wherein transcript stability is decreased without affecting NLRP3 promoter activity. We also demonstrate that azithromycin decreases NF-κB activity in human monocytes thereby illustrating at least two mechanisms by which the antibiotic negatively impacts IL-1β expression.

Our findings of decreased IL-1β expression in azithromycin-treated monocytes are consistent with observations of others [19, 20, 22]. However, decreased NF-κB activity has not consistently been seen after azithromycin treatment in all experimental systems. In fact, many studies that have shown inhibition of IL-1β expression by azithromycin have failed to show an effect on NF-κB activity [20, 22]. This difference may be explained by use of different monocyte cell lines or different methods of NF-κB activity measurement. However, it could also be explained by the presence of more than one mechanism of azithromycin-mediated IL-1β inhibition. In fact, in a murine model of LPS-induced pulmonary neutrophilia, decreased IL-1β expression was associated with decreased expression of another pro-inflammatory transcription factor in alveolar macrophages, activator protein-1 (AP-1). This is in keeping with our findings that clearly demonstrate more than one pathway of IL-1β inhibition, via decreased NF-κB activity and decreased NLRP3 mRNA expression. Here, using THP-1 Lucia cells containing a NF-κB responsive element upstream of a luciferase gene, we observed decreased NF-κB activity in both unstimulated and LPS-stimulated monocytes.

The inhibitory effect of azithromycin on inflammasome activity has also been previously shown [22]. However, in this study the mechanism by which azithromycin inhibits the inflammasome activity was via decreased caspase-4 induction [22]. Our studies are unique in that NLRP3 mRNA expression is decreased in LPS-stimulated monocytes treated with azithromycin. The observation that NLRP3 transcript stability is decreased was supported both by azithromycin transcript decay studies and experiments using cells transfected with the 3’UTR luciferase reporter experiments. There was no effect on NLRP3 promoter activity providing evidence against decreased NLRP3 mRNA synthesis as a mechanism. Importantly, these results imply multiple molecular regulatory actions by azithromycin depending on the cellular context and experimental design. In this regard, although azithromycin reduced 3’UTR luciferase reporter activity in our studies, its effect was not complete suggesting that other regulatory mechanisms may also be important for the transmission of this complex inflammatory signal. Given the previous report of decreased caspase-4 induction in monocytes treated with azithromycin, it appears that azithromycin exerts multiple inhibitory actions on inflammasome function.

The abundance of mRNA in a cell available for protein synthesis is a function of both its rate of production and its rate of degradation in the cytoplasm [25]. Transcript levels encoding individual proteins are degraded in cells by deadenylation in a complex process that involves specific enzymes [25]. Both intrinsic and extrinsic stimuli can activate signal transduction pathways that affect the stability of individual transcripts thereby allowing cells to respond rapidly in terms of altering protein synthesis [25]. The best characterized mode of mRNA stability regulation involves AU-rich elements (AREs) found in 3’UTRs of many labile transcripts whereby ARE-binding proteins (ARBPs) can associate [25]. The binding of ARBPs to the mRNA can result in either decreased degradation or accelerated decay of mRNAs [25]. Numerous upstream signaling pathways have been linked to regulate mRNA transcript rates of decay in this manner. For example, activation of the p38 mitogen-activated protein kinases (MAPKs) in macrophages exposed to LPS stabilizes both IL-1β mRNA and tumor necrosis factor alpha (TNFα) mRNAs to allow for initiation of an innate immune response [26]. CXC ligand 1 (CXCL1 or KC) mRNA is also stabilized by LPS in macrophages and this stabilization is antagonized by the presence of IL-10 in an ARE dependent fashion [27]. Additionally, mRNA degradation plays a significant role in the expression of many genes regulated by phosphatidyl 3-kinase signaling [28]. It is also well established that the actions of several commonly used antibiotics (macrolides, tetracyclines, and aminoglycosides) target prokaryotic RNA-metabolizing processes. While tetracycline is known to stabilize mRNAs within some bacteria [29], the results here demonstrate ability of azithromycin to modulate a host transcript intimately involved in the inflammatory response. Additional studies are needed to dissect the molecular signatures within the 3’UTR and RNA binding apparatus that regulates NALP3 mRNA lifespan in cells.

There are several caveats to our studies. While we have confirmed the effect of azithromycin on NALP3 levels in primary human peripheral blood mononuclear cells, our experiments were performed in vitro, and we have used concentrations of azithromycin that exceed the plasma concentration that has been measured in patients treated with this macrolide [30]. Nevertheless, these factors do not negate the observed effect of azithromycin on the inflammasome pathway which provides rationale for further work to validate these findings in experimental animal models and in humans.

## Conclusions

In summary, we show here for the first time that azithromycin not only decreases NF-κB activity and IL-1β expression in human monocytes, but also destabilizes NLRP3 transcript implicating both inflammasome dependent and independent mechanisms for downregulating IL-1β-mediated inflammation. These results suggest that there are multiple mechanisms by which azithromycin modulates inflammation which may provide new insight into potential molecular targets for antibiotic activities that foster the anti-inflammatory response.
